# Is the Experience of Thermal Pain Genetics Dependent?

**DOI:** 10.1155/2015/349584

**Published:** 2015-01-28

**Authors:** Emilia Horjales-Araujo, Joergen B. Dahl

**Affiliations:** Department of Anesthesia, Center of Head and Orthopedics, Copenhagen University Hospital, No. 4231, Blegdamsvej 9, 2100 Copenhagen, Denmark

## Abstract

It is suggested that genetic variations explain a significant portion of the variability in pain perception; therefore, increased understanding of pain-related genetic influences may identify new targets for therapies and treatments. The relative contribution of the different genes to the variance in clinical and experimental pain responses remains unknown. It is suggested that the genetic contributions to pain perception vary across pain modalities. For example, it has been suggested that more than 60% of the variance in cold pressor responses can be explained by genetic factors; in comparison, only 26% of the variance in heat pain responses is explained by these variations. Thus, the selection of pain model might markedly influence the magnitude of the association between the pain phenotype and genetic variability. Thermal pain sensation is complex with multiple molecular and cellular mechanisms operating alone and in combination within the peripheral and central nervous system. It is thus highly probable that the thermal pain experience is affected by genetic variants in one or more of the pathways involved in the thermal pain signaling. This review aims to present and discuss some of the genetic variations that have previously been associated with different experimental thermal pain models.

## 1. Introduction

The expression of pain is determined by a mosaic of neurobiological, cultural, and emotional factors [1–3], and the variability of pain responses between individuals is prominent [4]. However, previous studies have reported strong associations between pain ratings to, for example, thermal stimuli and activation of pain-related brain areas (e.g., the somatosensory cortex) [5, 6]; further, genetic variations have been shown to explain a significant portion of the variability in the expression of pain [7].

Studies of the genetic contributions to experimental pain perception can be highly relevant to clinical pain states. Thus, sensitivity to experimental pain has been suggested to predict acute and chronic pain states (e.g., [8–11]), and various studies have demonstrated that preoperative thermal pain scores may be associated with, for example, postoperative pain [12].

Thermal pain sensation is complex with multiple molecular and cellular mechanisms operating alone and in combination within the peripheral and central nervous system [13]. It is thus highly probable that the thermal pain experience is affected by genetic variants in one or more of the pathways involved in the thermal pain signaling.

The relative contribution of the different genes to the variance in clinical and experimental pain responses remains unknown [14]. Preclinical evidence, as well as gene association studies in animals and humans, suggests that the genetic contributions to pain perception vary across pain modalities [15–17]. For example, it has been suggested that more than 60% of the variance in cold pressor responses can be explained by genetic factors; in comparison, only 26% of the variance in heat pain responses is explained by these variations [17]. Thus, the selection of pain model might markedly influence the magnitude of the association between the pain phenotype and genetic variability.

A number of reviews have tried to highlight the association between genes and pain (chronic, acute, and experimental). Since the relation between genetic variations and pain seems to be dependent on the nociceptive model, a more detailed description of the genetic variations involved in each pain model is needed. The aim of this topical review is to present and discuss some of the genetic variations that have previously been associated with different experimental thermal pain models.

## 2. Methods

Reports pertinent to genetic variations associated with experimental thermal pain models were systematically sought using the PubMed database and Google Scholar without language restriction. Reports were considered for inclusion if they were carried out in healthy volunteers and published from 2000 to date. Free text combinations including the following search terms were used: “Polymorphism and thermal pain,” “polymorphism and cold pain,” “polymorphism and hot pain,” “polymorphism and pain,” and “polymorphism and hyperalgesia.” Reference lists from retrieved articles were searched for additional papers. The last search was performed on December 15, 2013.

A synopsis of the results is presented in Supplementary Table 1 available online at http://dx.doi.org/10.1155/2014/349584.

## 3. Transient Receptor Potential Channels

**Figure 1:**
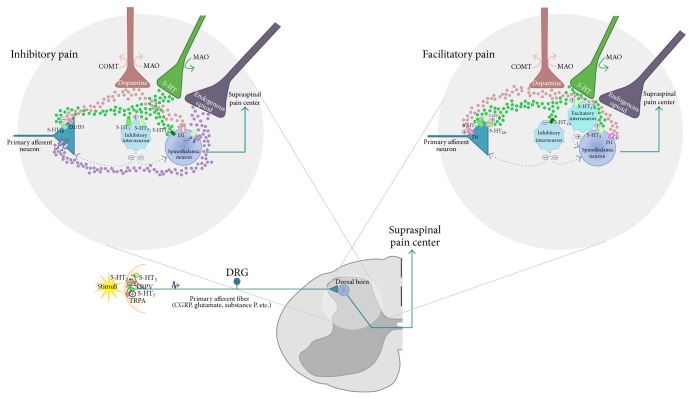
Peripheral and central modulation of the nociceptive signal. Serotonin receptors as well as transient receptor potential (TRP) channels in the periphery are activated in response to a nociceptive stimuli and enable the generation of an action potential. Signals will be propagated through the primary afferent neuron reaching the first synapse at the dorsal horn of the spinal cord. Centrally the nociceptive signal is controlled by descending pathways. Inhibitory descending serotonin released in the dorsal horn can either bind with serotonin receptor 1_B_ located in the primary afferent neuron inhibiting the first synapse; bind to serotonin receptor 7 and/or 3 activating the inhibitory interneuron; or bind with serotonin receptor 1_A_ in the spinothalamic neuron. The nociceptive signal can be also inhibited by binding of descending dopamine with its receptor D2/D3 in the primary afferent and/or in the spinothalamic neuron or by binding of opioids with receptors *δ* and *μ* in either the primary afferent or in the spinothalamic neuron. Pain signals can also be facilitated by central descending pathways, in which serotonin can excite the primary afferent neuron by binding with serotonin receptors 7, 3, and 2_A_. Serotonin can also bind with receptor 1_A_ inhibiting the inhibitory interneuron or with serotonin receptor 3 located either in the excitatory interneuron or in the spinothalamic neuron, enhancing the nociceptive signal. Dopamine has also a facilitatory effect by binding with receptor D1 located either in the primary afferent or in the spinothalamic neuron. MAO and COMT can participate in the inhibitory and facilitatory nociceptive signaling by regulating the degradation of either serotonin or dopamine in the presynaptic neuron.

Transient receptor potential (TRP) channels are a group of ion channels involved in sensory systems responding to temperature, touch, and osmolality as well as to painful stimuli (see Figure 1). TRP channels are divided into seven subfamilies. The TRP subfamily A member 1 (TRPA1), expressed in, for example, the dorsal root ganglion, trigeminal ganglion, and hair cells, is activated by noxious cold temperatures (<15°C) [18]. Encoded on the chromosome 8 is the* TRPA1* gene which includes an intronic rs11988795 polymorphism that has been associated with cold pain perception. Thus, individuals who are homozygous for the G-allele have been reported to have longer cold-withdrawal time than those with the AA genotype [19].

Another TRP subfamily V, expressed mostly in central nervous system (CNS), is proposed to mediate chemical and hot thermal noxious heat sensations (>43°C and capsaicin) [20]. TRPV1 is localized on chromosome 17 and consists of 16 exons [21]. The rs8065080 polymorphism is predicted to reside within membrane-spanning helix 5 and affects the transmembrane domain, which confers responsiveness to capsaicin (chili peppers). This SNP consists of an G>A substitution, resulting in an isoleucine to valine change at codon 585; female European Americans homozygous for the isoleucine-allele showed longer pain response time to cold and heat stimuli [22]. Curiously, these results were not replicated in a following study from the same research group [19].

## 4. Opioids Receptors

Opioid receptors are a group of G protein-coupled receptors essential for morphine and other opioids to consolidate their analgesic effect (see Figure 1). These receptors are expressed in multiple brain regions, in the periphery, and on various immune cells [23, 24]. Three opioids receptors families have been studied in relation to thermal pain perception.

Morphine, a strong analgesic, produces its analgesic effects primarily by acting as an agonist at the *μ* opioid receptor [25]. The human *μ* opioid receptor is encoded in the* OPRM1* gene, located at chromosome 6, and is mostly expressed in the CNS (e.g., dorsal horn of the spinal cord, periaqueductal gray region, nucleus accumbens, amygdala, and cerebral cortex) and in the intestinal tract [26]. One of the most studied polymorphisms in this gene is the rs1799971, also known as A118G. This missense variation generates an amino acid exchange from asparagine to aspartate which reduces opioid receptor signaling efficiency and expression. This SNP has been associated with decreased opioid potency, suggesting that increased doses of the drug might be required in selected patients [27]. Fillingim and colleagues found that male carriers of the rare allele reported lower pain ratings delivered by a computer-controlled thermode to raise the temperature at a rate of 0.5°C/s, while women with the same genotype reported higher pain ratings [28]. The authors therefore suggest that there is a gender and genotype interaction related to the heat pain ratings. In addition, an ethnic-dependent association has been suggested between this polymorphism and pain perception [29]. In this study, the authors reported that the G-allele was associated with decreased pain sensitivity among non-Hispanic whites; interestingly a trend in the opposite direction was observed in Hispanics. In a more recent study, no association of this polymorphism with either thermal pain generated by a thermode (32°–52°C at increasing rate of 1°C/s) was observed in Caucasian volunteers experiencing experimental pain [30]. If the effect of this polymorphism on pain perception is indeed gender and/or ethnic dependent, a more precise description (avoiding broad-based social constructions) of the ethnicity of the participants is required to be able to reach a conclusion.

In humans, only few studies investigate the influence of polymorphisms in opioid receptor *κ* (OPRK) on pain sensitivity [30]. This receptor has dynorphin as the primary endogenous ligand [31]; it has thus been linked to analgesia, anticonvulsion, diuresis, and dysphoria. Widely expressed on the brain and spinal cord, the* OPRK* gene is encoded in chromosome 21 and contains the rs643799 polymorphism. This intronic polymorphism consists of a T>C substitution. When studied in experimental conditions, Caucasian C-carriers seem to present lower thermal pain thresholds in comparison with T-allele homozygous [30], although this result should be taken with some precaution since the association was just outside significance after correction for multiple comparisons.

Probably one of the most experimentally studied opioid receptors is the opioid receptor *δ* (OPRD). The opioid receptor *δ* has been suggested to play a role in mechanical, neuropathic, and inflammatory pain [32]. In a study of 500 healthy participants, a polymorphism in* OPRD* rs2234918 was associated with a gender-specific difference in thermal pain sensitivity when pain was generated by applying a thermode to the forearm with increasing temperature at 5 s rate [22]. However, following studies from the same researcher [19] and other researchers [30, 33] failed to find any association between the OPRD polymorphism alone and an OPRD haplotype containing this polymorphism.

Similar results have been observed when the rs1042114 polymorphism in the same gene was studied in relation to thermal pain sensation. The rs1042114 polymorphism results in a cysteine to phenylalanine substitution. Although in a first study Kim and colleagues had reported a marginal significant decrease in heat pain intensity associated with this polymorphism [22], more recent studies have failed to find any association between the mentioned polymorphism and thermal (neither heat nor cold) pain in a Caucasian sample [30, 33].

## 5. Serotonin Transporter and Receptors

Serotonin is a neurotransmitter that has been suggested to play a role in mood and pain regulation (see Figure 1). A key player in serotonin (5-HT) signaling is the serotonin transporter (5-HTT), which regulates the uptake of 5-HT into the presynaptic neurons for recycling or degradation after serotonin has been released, thus playing a critical role in determining the duration and intensity of serotonin communication. The serotonin transporter is coded by a single gene (*SLC6A4*) located on the long arm of the chromosome 17 [34]. The protein has been found to be largely expressed in the CNS (raphe nuclear complex, amygdala, thalamus, hypothalamus, substantia nigra, and locus coeruleus) and in addition, it has also been suggested that 5-HTT is localized in the periphery, for example, intestinal tract, placenta, lungs, and blood platelets [35]. A well-described polymorphism in the promoter region of the gene (5-HTTLPR) appears to influence the efficiency with which the 5-HTT returns serotonin to the presynaptic neuron by affecting the expression of the gene [36]. This polymorphism consists of a 44 bp insertion/deletion in a C/G-rich variable number tandem repeat sequence located in the promoter region of the* SLC6A4 *gene, resulting in long (L) and short (S) arms. The S-allele is coupled to reduced gene expression, leading to lower densities of the serotonin transporter [37, 38]. This polymorphism is coupled with a single nucleotide polymorphism (rs25531) also located on the promoter region of the gene, which is an A/G substitution. The G-allele is mostly linked to the L-allele of the 5-HTTLPR and has been shown to reduce the transcriptional efficacy to the level of the S-allele [39, 40]. The combination of these two polymorphisms is referred to as the triallelic 5-HTTLPR and permits a functional division of the individuals into having high, intermediate, or low expression of the 5-HTT.

Some studies have identified an association between the triallelic polymorphism and both muscular [41, 42] and thermal [43] pain modulation. Nevertheless, studies on the association between the polymorphism and thermal pain thresholds are inconclusive. On one hand, a study reports no relationship between the (biallelic) 5-HTTLPR and heat-pain thresholds [44]. However, Lindstedt and colleagues grouped 44 healthy European descent individuals based on their triallelic 5-HTTLPR polymorphism. The authors observed that thresholds to thermal pain sensitivity are associated with the triallelic 5-HTTLPR. Thus, the low 5-HTT expressing group exhibited significantly reduced sensitivity to heat and cold pain when compared to the high 5-HTT expressing group [45]. Nevertheless both studies included a low number of participants and the association between the triallelic 5-HTTLPR and thermal pain threshold remains uncertain. Interestingly, animal studies have also reported that knockout mice for the 5-HTT present reduced thermal hyperalgesia [46, 47], supporting the hypothesis of a correlation between polymorphism in the 5-HTT and thermal pain.

To our knowledge only one more polymorphism in this gene has been studied in relation to thermal pain perception; however, no associations were found [48].

Only few serotonin receptors have been investigated in relation to experimental thermal pain. Lindstedt and colleagues, for example, studied the rs6295 in the serotonin receptor 1A (*5HTR1*_*A*_). The authors reported that G-carriers with European decent exhibited a significant reduced sensitivity to cold pain (lower thresholds) and a tendency (although not significant) for higher heat-pain thresholds [49].

## 6. Dopamine Transporter and Receptors

Research indicates that dopamine plays a role in pain perception [50]. The dopamine transporter (DAT) is a key modulator of dopamine transmission. Expressed mostly on the CNS and encoded in gene* SLC6A3*, the DAT mediates the reuptake of the neurotransmitter from the synaptic cleft to the presynaptic neuron. An insertion/deletion of 40 bp in the 3′-untranslated region has been found to be associated with headache [51] and for that reason has been further studied in experimental conditions. Treister and colleagues observed an association between this polymorphism and tolerance to cold pain [52], but interestingly no association was observed between this polymorphism and conditioned thermal pain inhibition [42]. These data suggest that DAT is involved in the sensory component of cold pain tolerance, rather than the modulatory component of it.

Some dopamine receptors have been studied in relation to thermal pain perception. Dopamine receptor 3, for instance, with major expression in the nucleus accumbens is encoded in the* DRD3* gene and contains the rs6280 polymorphism [53]. This polymorphism is also known as Ser9Gly because the G>A substitution results in an amino acid exchange of glycine > serine. It has been reported that Gly-allele homozygous (linked to highest receptor activity) presents higher thermal pain thresholds in both healthy participants and fibromyalgia patients [54].

The dopamine receptor 4 has also been studied in relation to thermal pain perception. Thus, Treister and colleagues have studied a polymorphism consisting of 48 bp insertion/deletion in the exon 3 of the gene encoding for the dopamine receptor 4 (*DRD4*). The authors did not observe any association between the polymorphism and either cold or heat noxious stimuli [52]. In addition, no association was observed between the genetic variation and conditioned thermal pain modulation [42].

## 7. Catechol-O-Methyltransferase

Probably one of the most extensively studied pain candidate genes is catechol-O-methyltransferase (*COMT*) gene. This gene is located on the chromosome 22 and encodes the COMT enzyme. The enzyme has higher expression in the CNS and the liver and degrades catecholamines such as dopamine, adrenaline, and noradrenaline [55]. In general, low COMT activity has been related to increased pain sensitivity.

A well-known variation in the* COMT* gene, a G/A substitution polymorphism (rs4680, also called Val158Met), produced a substitution of valine to methionine. This polymorphism is the only nonsynonymous SNP in the* COMT* gene and alters the enzyme stability and activity to approximately 20–40% in Met-homozygous [56, 57]. In an* in vitro* study, Diatchenko and colleagues observed that reductions in COMT enzymatic activity enhance pain sensitivity and inhibition of COMT in rats resulted in a profound increase in pain sensitivity [58]. A great variety of clinical studies have associated this genetic variation with pain perception, suggesting that the genotype encoding Met/Met may predispose for chronic pain conditions and psychological outcome in patients with pain, for example, [59–61]. However, some data is quite contradictory and several studies have failed to show a genetic association with postsurgical pain [62] and chronic widespread pain [63–65]. Although these polymorphisms have been reported to be associated with experimental muscle pain (generated by hypertonic solution) [59], results from experimental thermal pain studies are also contradictory. On one hand, Diatchenko and colleagues were able to observe greater heat pain temporal summation in Met-homozygous [58]. In addition, COMT Val158Met genotype has been observed to markedly influence the pain experience following standard heat pain provocations in the period following an opiate injection [66]. Nevertheless the majority of experimental studies have failed to find significant associations between this polymorphism and either cold [19, 67, 68] or heat [33, 68, 69] pain perception.

It has been suggested that the effect of rs4680 may depend on the genomic environment. Thus, haplotypes studies (combinations of more than one SNP) have reported that a haplotype conformed to rs6269, rs4633, rs4818, and rs4680 is associated with lowest pain sensitivity to mechanical and thermal pain [58]. Furthermore, the carriers of the GCGG-allele combination have been classified as “low pain sensitive” and have the least pain responsiveness to ischemic, mechanical, and thermal stimuli [70]. It is, thus, possible that some SNPs affect thermal pain perception by interacting with other SNPs.

Similar to rs4680, inconsistent results have been reported when thermal pain perception is studied in relation to other polymorphisms in the* COMT* gene. For example, some studies have reported no association between the synonymous rs4633 polymorphism and thermal pain in a Caucasian sample [33], while others have shown that this polymorphism is related to decrease in thermal pain perception [58].

Other less studied polymorphisms have also been reported to have some association with thermal pain perception, for example, rs6269 [58], rs165599 [71], and rs4646312. In addition, a more recent study genotyping 22 different* COMT* gene SNPs in woman undergoing breast cancer surgery reported that the 3′-UTR SNP rs887200 and the intronic rs165774 polymorphism, whose effects on pain have not been described before, showed the strongest evidence of association with thermal pain sensitivity [68]. Even though there is evidence supporting the hypothesis of a role of the* COMT* gene in thermal pain perception, these results need to be replicated.

## 8. Monoamine Oxidase

Monoamine oxidases (MAO) are flavin-containing mitochondrial enzymes that catalyze the oxidative deamination of neurotransmitters and biogenic amides in the brain and in the peripheral tissue. There are two isoforms of the enzyme that differ significantly in their substrate specificity and regulation [62]. Although both can be found in both CNS and the periphery (e.g., heart, liver, blood vessels, and kidney) [72], typically, MAO-A catalyzes the oxidation of serotonin, whereas MAO-B acts on 2-phenylethylamine and benzylamine.

Located on the X chromosome, the* MAO-A* gene contains a 30 bp insertion/deletion polymorphism in the promoter region. In addition to the association between this polymorphism and postoperative pain ratings [62], Treister and colleagues reported an association between this genetic variation and tolerance to pain generated by the cold pressor test (ice water at 1°C) [52].

## 9. Fatty Acid Amide Hydrolase

The fatty acid amide hydrolase (FAAH) is the enzyme that terminates the endogenous signaling activity of a large class of fatty acid amides, for example, anandamide. It is suggested that decreased FAAH expression or net activity causes higher synaptic availability of anandamide increasing its activity as pain defense mechanism; similarly, a rise of FAAH activity might increase pain.

Two intronic FAAH variants (rs4141964 and rs2295633) have been associated with increased cold pain perception generated by submersion of the hand into 2–4°C water [19]. In addition, Chiang and colleagues have reported that the rs324420, a nonsynonymous polymorphism generating a proline > threonine substitution, is associated with variability in the endocannabinoid system which may influence pain sensitivity [73].

## 10. Melanocortin-1 Receptor

The melanocortin-1 receptor (*MC1R*) gene is best known for its role in skin and hair pigmentation and is expressed on the plasma membrane of melanocytes. Animal studies have reported that mice (C57BL/6-Mc1r (e/e)) with nonfunctional MC1Rs exhibit a consistently decreased sensitivity to pain across a broad range of nociceptive modalities [74]. Similar results have been observed in humans [74]. The rs1805007 polymorphism, also known as Arg151Cys, is well known for conferring the red hair color. Interestingly, it has been reported that redheaded women (T-allele homozygous) are more sensitive to thermal pain stimuli than women carriers of the C-allele exposed to a thermode with temperature increasing rate of 0.5°C/second [75].

## 11. Considerations and Conclusions

This review has summarized the existing evidence for associations between polymorphisms and variability in thermal pain perception. Contradictory with our expectations, only few polymorphisms have been consistently associated with thermal pain perception (e.g.,* TRPA1 *gene). Research is, however, far from identifying all genetic variations associated with pain. Most of the studies on the genetics of pain have either failed to be replicated or have only been partially replicated. This is mostly due to the substantial limitations of the studies. For instance, variability in experimental pain methodology might lead to contradictory results. In addition, a variety of studies included small populations, leading to possible underpowering, which are closely connected with another frequent mistake, that is, inappropriate choice of statistical analysis. For example, a variety of studies do not correct for multiple comparisons, leading to type 1 errors (incorrect rejection of a true null hypothesis), thus presenting false positive results.

Additionally, studies on the genetics of pain often present design problems, either by having genotyping errors and poor phenotyping or by including heterogeneous populations (including different ethnicities or not even correcting for possible gender differences).

Furthermore, only few studies take into account gene x gene interactions. In this aspect, it is important to mention that in most cases multiple genetic variations result in a disposition to a particular disease and each variant makes a small contribution to the overall susceptibility to pain. Thus, the inclusion or exclusion of interactions between SNPs might affect the phenotype observed, hindering the replication of results. Thermal pain studies considering gene x environment interactions are also of significant importance. In a more recent study, Bell and colleagues analyzed the epigenetic changes (methylation) on pain-related genes [76] in both identical twins and unrelated individuals. The authors reported a strong association between the* TRPA1* gene and thermal pain. Further studies following this line of methodology would be of great relevance in the pain genetic field.

Although scarcely studied, genetic variations in the serotonin pathways are promising. Serotonin is involved in both peripheral and central mechanisms of pain neurotransmission [77]. Although no conclusive results in relation to cold pain nociception have emerged [48], genetic variations in the serotonin transporter gene have been associated with heat pain perception. 5-HTT-knockout mice exhibit markedly reduced thermal hyperalgesia in a model of neuropathic pain [46, 47]. With one exception [44], studies in humans have associated variations in the* SLC6A4* gene with heat pain perception in chronic pain patients [78] and in healthy participants [45]. In addition, genetic variation in the serotonin receptor 1A has also been associated with thermal pain perception [49]. Furthermore, 5-HTT is also involved in mood regulation, and genetic variations in this gene [43, 79] and other serotonin genes [80] have been associated with emotional aspects of the pain experience.

Serotonin receptors have also been suggested to play a role in secondary hyperalgesia in animal models (e.g., [81–83]). However, there are no studies on the relation between serotonin polymorphisms and hyperalgesia in humans. Since there is a general lack of studies on the relation between polymorphisms and thermal hyperalgesia in healthy participants, further studies are highly encouraged to pursuit this area.

As whole genome sequencing has become more cost efficient, it is now easier to examine the effect of genetic variants on pain phenotypes. However, it has to be done in a standardized and systematic way, minimizing differences in methodologies and data analysis between studies, to achieve a better understanding of the relation between pain and genetic variations. Since experimental pain sensitivity is known to be a predictor for pain pathologies, better understanding of the genetics behind experimental pain might have an important contribution to the understanding of clinical pain states. Thus, increased understanding of pain-related genetic influences may identify new targets for therapies and treatments.

## Supplementary Material

Supplementary Table: Summary of single nucleotide polymorphism (SNP) previously related with thermal pain perception and analyzed in this review.
