# Supplementary material for: Is the Experience of Thermal Pain Genetics Dependent?
Source: Biomed Res Int. 2015 Jan 28;2015:349584. doi: 10.1155/2015/349584 (PMC4324494; doi:10.1155/2015/349584)
Supplement: Supplementary file 1 — Supplementary Table: Summary of single nucleotide polymorphism (SNP) previously related with thermal pain perception and analyzed in this review. [file 349584.f1.pdf]

gene	full name	chromosome	polymorphism (dbSNP database number)	minor/major allele	position	relation with pain	reference	metod
TRPA1	Ankyrin like protein	*11*	rs1198795	G/A	intronic	A/A genotype reported less cold pain tolerance compared to G/G (mixed ethnicities)	Kim et al., 2006	ice cold water
TRPV1	Vanilloid Receptor Ionophore	17	rs8065080	C/T(isoleucine)	exonic (missense)	females European Americans T/T showed longer pain-response time to cold stimuli.	Kim et al., 2004	ice cold water
						not significant association with cold pain sensitivity in Europeans Americans	Kim et at.,2006 and 2012	ice cold water
OPRD1	Opioid Receptor δ1	1	rs2234918	C/T	exonic (synon)	increased heat pain intensity	Kim et al., 2006	thermode 35 to 49°C at 5s
						not association with variation in thermal pain thresholds	Sato et al., 2013	thermode at 1s
						no difference in pain perception between genotypes in Caucasian sample	Doehring et al., 2011	thermode at 3s
			rs1042114	G/T(Phe)	exonic (missense)	marginal significant decrease in heat pain intensity in heterozygous.	Kim et al., 2004	thermode 35 to 49°C at 5s
						not association with variation in thermal pain thresholds in Caucasian sample	Sato et al., 2013	thermode at 1s
						not association with pain thresholds: von frey, heat, cold, blunt pressure either electric in Caucasian sample	Doehring et al., 2011	thermode at 3s
COMT	cathecol-O-methyltransferase	22	rs4680 (Val158Met)	G/A (Met)	exonic (missense)	no association with cold pressor pain	Birklein et al., 2008	cold pressor test
						greater heat pain temporal summation VAS reported in AA genotype	Diatchenko et al., 2005	computer controlled thermode masseter muscle skin
						no association with heat/cold pain sensation in Europeans Americans	Kim et al., 2006	thermode 35 to 49°C at 5s
						no difference in capsasin heat pain effect sizes between genotype in Caucasian sample	Doehring et al., 2011	thermode at 0.3°/s
						no genotype influence on heat pain perception neither in healthy subjects nor in borderline personality disorder. Ethnicity not specified	Schmahl et al., 2012	peltier based thermode

			rs4633	C/T	exonic (synon)	no difference in pain effect sizes between genotype in Caucasian sample	Doehring et al., 2011	thermode at 0.3°/s
						decreased thermal and pressure pain	Diatchenko et al., 2005	computer controlled thermode masseter muscle skin
						heterozygous presented higher pain threshold	Xiang et al, 2012	
			rs6269	G/A	intronic	accounted for 6% of the variation in pain sensitivity	Diatchenko et al., 2005	
			rs4818	G/C/T	exonic (synon)	accounted for 7% of the variation in pain sensitivity	Diatchenko et al., 2005	
			rs4646312	C/T	promoter	decreased cold pain intensity	Kim et al., 2006	
			rs165599	G/A	3/A65	associated with thermal pain ratings to the initial first pulse from the train of 10 pulses delivered at 47 °C in Caucasians females	Belfer et al., 2013	
DRD3	dopamin receptor d3	3	rs6280(Ser9Gly)	C/T (Ser)	exonic (missense)	Gly-Gly presented higher thermal pain thresholds in both healthy participant and fibromyalgia patients. Gly-Gly (linked to highest receptor activity) was associated with higher TPTs, that is less analgesia	Potvin et al, 2009	thermode with temperature increasing from 32 to 51 by a rate of 0.3 degrees per second
SLC6A3 (DAT)	Dopamine Transporter	5	40bp repeat in the 3´untranslated region		30bp	No association of condition thermal pain inhibition	Treister et al., 2011	cold pressor test conditioned modulation.
						association between this polymorphism and tolerance to cold pain	Treister et al., 2009	cold pressor thest with water bath at 1°C
MC1R	Melanocortin Receptor 1	16	rs1805007 (Arg151Cys)	T/C (Cys)	exonic (missense)	redheaded woman (Arg) are more sensitive to thermal pain stimuli.	Liem et al, 2005	exposed to a thermodein the forearm with temperature increasing rate of 0.5°C/second [37].
SLC6A4	serotonin transporter	17	5-HTTLPR	Sa/Lg/La	promoter	high expression group presented lower heat pain threshold and higher cold-pain threshold when compared to low expression. (low expression group showed reduced sensitivity to heat and cold pain) in self-reported European decent.	Lindstedt et al., 2011	thermode

						relative to the s-allele homozygous, the carriers of the l-allele have an additively higher thermal pain inhibition. OBS: only bi-allelic was genotypes and also, mixed ethnicities.	Treister et al., 2011	thermode to the hand 37-47 at 10degrees per second
			rs3813034	A/C	3/C81	no differences in pain thresholds (cold and pressure pain) between genotype in Japanese population.	Aoki et al., 2010	cold pressor test
5-HTR1A	serotonin receptor 1A	5	rs6295	C/G	promoter	G-carriers of European decent exhibited the expected thermal hypoalgesia at threshold-level intensities, as compared to C-homozygote (perhaps hypoalgesia was abolished at higher intensities). G-carriers exhibited reduced sensitivity to cold pain (lower thresholds) and a tendency for higher heat-pain thresholds, but not significant, perhaps underpowered problem.	Lindstedt et al., 2012	A computer controlled Peltier-type thermode
OPRM1	Opioid Receptor µ1	6	rs1799971	G/A(Asn)	exonic (missense)	significant interaction between sex and genotype in heat pain ratings at 49C. The rare allele was associated with lower pain ratings among men but higher pain ratings among women.	filligim et al., 2005	thermode in 3 different parts of the body 0.5C/second
						No association with either thermal pain or muscle pain in Caucasians volunteers	Sato et al., 2013	computer driven heat pain device (thermode) in forearm 32-52 at 1C/s
OPRK	opioid receptor K	21	rs643799	T/C	intronic	C-carriers presented lower thermal pain thresholds	Sato et al., 2013	computer driven heat pain device (thermode) in forearm 32-52 at 1C/s
